# Unravelling the structural plasticity of stretched DNA under torsional constraint

**DOI:** 10.1038/ncomms11810

**Published:** 2016-06-06

**Authors:** Graeme A. King, Erwin J. G. Peterman, Gijs J. L. Wuite

**Affiliations:** 1 Department of Physics and Astronomy and LaserLaB Amsterdam, Vrije Universiteit Amsterdam, De Boelelaan 1081, 1081 HV Amsterdam, The Netherlands

## Abstract

Regions of the genome are often held under torsional constraint. Nevertheless, the influence of such constraint on DNA–protein interactions during genome metabolism is still poorly understood. Here using a combined optical tweezers and fluorescence microscope, we quantify and explain how torsional constraint influences the structural stability of DNA under applied tension. We provide direct evidence that concomitant basepair melting and helical unwinding can occur in torsionally constrained DNA at forces >∼50 pN. This striking result indicates that local changes in linking number can be absorbed by the rest of the DNA duplex. We also present compelling new evidence that an overwound DNA structure (likely P-DNA) is created (alongside underwound structures) at forces >∼110 pN. These findings substantiate previous theoretical predictions and highlight a remarkable structural plasticity of torsionally constrained DNA. Such plasticity may be required *in vivo* to absorb local changes in linking number in DNA held under torsional constraint.

## Abstract (web summary)

Cellular DNA is often torsionally constrained, but the topologies that such DNA can adopt under tensile strain have remained largely untested. Here, the authors use single-molecule optical tweezers to illustrate the structural plasticity of torsionally constrained DNA under mechanical force.

## Graphical abstract

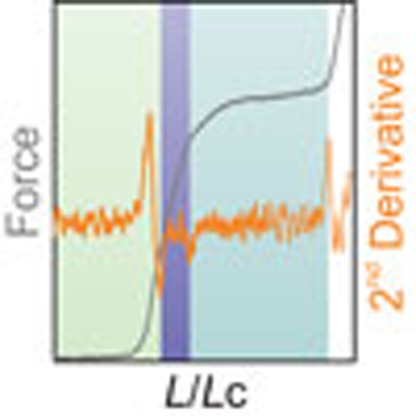

Torsional constraint facilitates regulation of the genome: DNA transactions are typically initiated by mechanical forces, applied by proteins, to sensitively tune the local structure of DNA^1^. Under torsional constraint, the sum of the twist (Tw) and the writhe (Wr) of the DNA molecule is a constant, known as the linking number (Lk=Tw+Wr). Here Tw describes the helical winding of the DNA strands around each other, while Wr is a measure of the coiling of the double-helix axis of DNA. Recently, an increasingly sophisticated array of single-molecule manipulation techniques has been developed to study the structural landscape of DNA under applied tension or torque in unprecedented detail^2^,^3^,^4^,^5^,^6^,^7^,^8^,^9^,^10^,^11^. However, it is still unclear how the local structure of DNA responds to mechanical force when Lk is fixed, particularly at tensions greater than a few pN.

To address this question, we examine the influence of torsional constraint on the structural integrity of DNA as a function of tension. Free from torsional constraint, DNA displays a striking interplay between twist and stretch: applied forces up to 30 pN induce slight overwinding of the double helix, while increasing the force further from ∼35 to 65 pN results in underwinding of the double helix^12^,^13^. The latter enables a small extension of the molecule of up to ∼10%. At ∼65 pN, a structural transition (overstretching) occurs whereby the molecule extends up to 70% over a narrow force range^14^,^15^. In torsionally relaxed DNA with closed ends, this transition is associated with the formation of underwound DNA structures (∼37.5 bp per turn) that are either basepair-melted (bubble-melted) or base-paired (S-DNA)^4^,^5^,^6^.

In the absence of pre-applied torque, torsionally constrained DNA overstretches at forces of ∼110 pN, significantly higher than for torsionally relaxed DNA^16^,^17^,^18^,^19^. A number of theoretical models have been developed that can largely reproduce the force-extension behaviour of torsionally constrained DNA^16^,^20^,^21^. These models predict that overstretching of torsionally constrained DNA (*F*≥110 pN) is associated with formation of S-DNA in combination with P-DNA, in a ratio of ∼80:20. P-DNA is an overwound form of DNA, where the backbones are tightly wrapped around one another, causing the basepairs to flip out. The predicted structure of P-DNA is ∼60–70% longer than B-DNA with an estimated twist of ∼2.5 bp per turn^16^,^22^,^23^. The combination of underwinding and overwinding was proposed to enable torsionally constrained DNA to extend by up to 70% during overstretching while conserving Lk^16^. However, to date there has been no direct confirmation that a specific structure of P-DNA is formed in torsionally constrained DNA as a result of applied tension.

Moreover, torsionally constrained DNA displays a significantly more gradual change in force as a function of extension than for torsionally relaxed DNA. Theoretical models predict that such ‘softening' of the force-extension curve in the case of torsionally constrained DNA is due to a limited formation of S-DNA at forces <110 pN (refs 20, 21). Thus far, however, this prediction has not been tested experimentally. Moreover, how this can occur in a twist-constrained DNA molecule is unclear, as is the role of ionic strength and basepair sequence. Using a novel approach of correlating 2nd derivative analysis of force–distance (FD) measurements with fluorescence imaging of a single-stranded DNA-binding protein, we explain the complex FD behaviour observed for torsionally constrained DNA. We show that DNA under torsional constraint has a surprising plasticity, which may be exploited *in vivo* as a means to absorb local changes in Lk.

## Results

### Two distinct tension-dependent structural transitions

**Figure 1:**
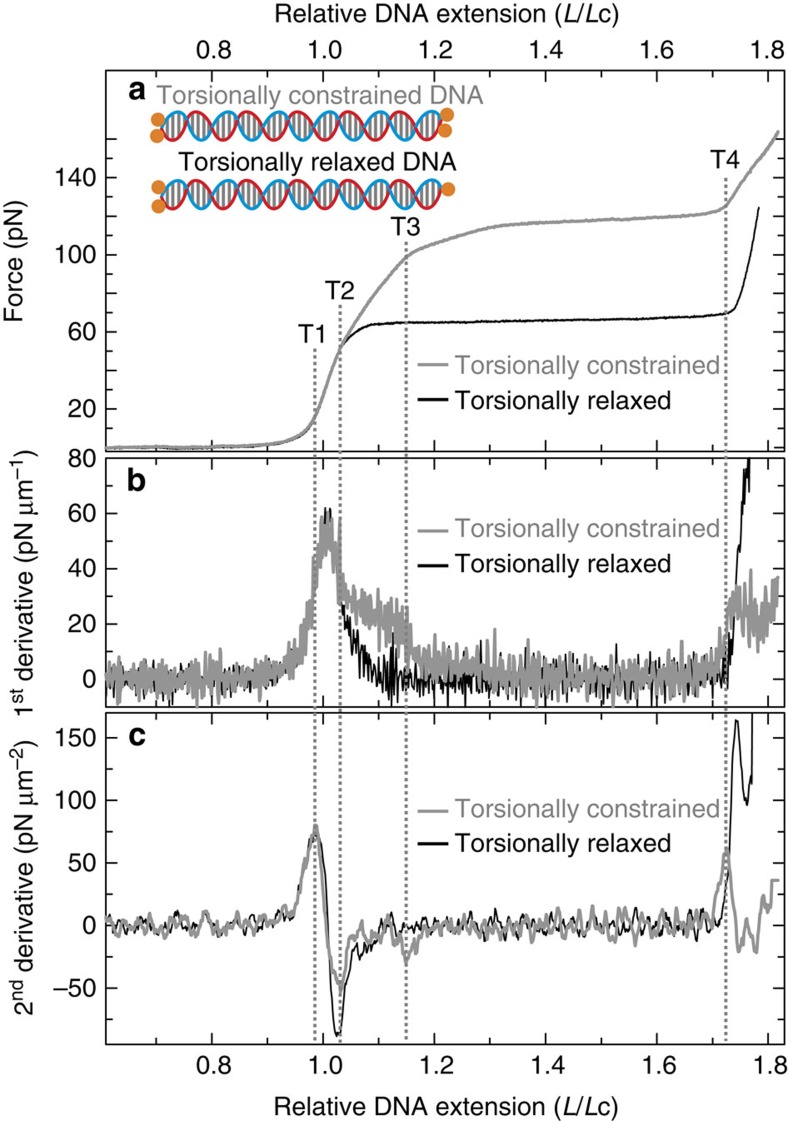
FD curves and their derivatives of torsionally constrained and torsionally relaxed DNA. (**a**) Average FD curves of torsionally constrained DNA (grey) and torsionally relaxed DNA (black) in 25 mM NaCl. Inset highlights the construct design for torsionally constrained DNA and torsionally relaxed DNA. In both cases, each end of the DNA molecule is ligated to an end-cap labelled with biotin moieties (orange). The DNA is only torsionally constrained if ≥2 biotins on each end of the molecule are bound to streptavidin-coated optically trapped beads. (**b**) First derivative of the FD curves displayed in **a**. (**c**) second derivative of the FD curves displayed in **a**, obtained using a Savitzky-Golay (20 point) smoothing filter. Dashed lines indicate where the four peaks (T1–T4) identified in **c** lie in the FD curves.

We first prepared end-capped DNA molecules (based on λ-DNA, ∼48.5 kb) with and without torsional constraint when bound to optically trapped beads. These construct designs are highlighted in Fig. 1a (inset) and shown in greater detail in Supplementary Figs 1 and 2. Average FD curves for each of the above DNA construct designs (in 25 mM NaCl) are shown in Fig. 1a. To quantify the curvature of FD curves of torsionally constrained DNA and end-closed torsionally relaxed DNA, we extracted the first and second derivatives of those FD curves displayed in Fig. 1a. These are plotted, for relative DNA extensions (*L*/*L*c) between ∼0.6 and 1.8, in Fig. 1b,c, respectively.

In the case of torsionally relaxed DNA, a prominent peak is observed in the first derivative plot at *L*/*L*c ∼1.01, consistent with a quasi two-state overstretching transition (Fig. 1b, black trace). This signal translates into two peaks in the second derivative profile. Each of these peaks coincides with a change in the FD curve that can be correlated with the expected mechanical properties of torsionally relaxed DNA. The first of these peaks (termed T1; *L*/*L*c ∼0.99) corresponds to the expected switch from entropic stretching to enthalpic stretching, while the second (termed T2, *L*/*L*c ∼1.03) is associated with the transition from enthalpic stretching into overstretching (Fig. 1c, black trace).

Similar behaviour is evident for torsionally constrained DNA (Fig. 1b,c, grey traces). Here, however, a shoulder (at *L*/*L*c ∼1.1) rests on the main peak in the first derivative plot, yielding an additional (T3) peak in the second derivative trace at *L*/*L*c ∼1.15. Furthermore, the intensity of the T2 peak in the second derivative plot for torsionally constrained DNA is around half of the value observed for torsionally relaxed DNA. The above analysis leads us to conclude that at least two structural transitions occur in torsionally constrained DNA as the applied force is increased (T2 and T3). This contrasts with torsionally relaxed DNA, where only one such transition is identified (T2). Finally, we note that a peak in the second derivative trace is also present at *L*/*L*c ∼1.73 for both torsionally constrained DNA and torsionally relaxed DNA. This peak is associated with the transition to enthalpic stretching of overstretched DNA, and for consistency, is labelled T4 in both cases.

### Ionic strength dependencies of second derivative transitions

**Figure 2:**
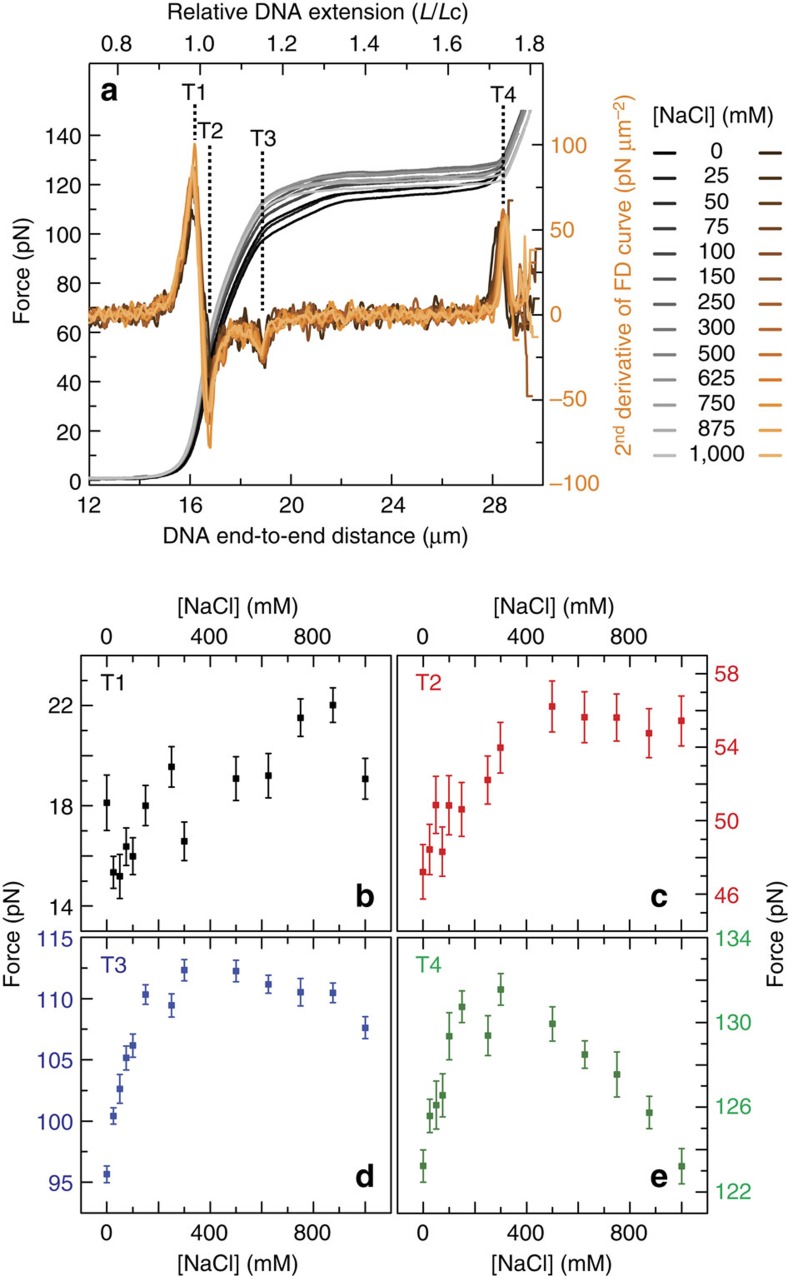
FD curves and their second derivatives of torsionally constrained DNA under varying ionic strength. (**a**) Average FD curves (grey-scale), together with the second derivative of each curve (orange-scale) as the concentration of NaCl is increased. Dashed lines highlight the peaks in the second derivative profiles associated with T1–T4, as defined in Fig. 1. (**b**–**e**) Force associated with torsionally constrained DNA at T1–T4, respectively, as a function of NaCl concentration. Errors are standard errors of the mean (s.e.m.; *N*∼10).

The energy barrier for a change in DNA structure is often modified by the ionic strength of the buffer solution^4^,^5^,^6^,^24^. To understand more about the structural transitions identified in Fig. 1, we, therefore, investigated whether, and how, T1–T4 vary as a function of monovalent (sodium) and divalent (magnesium) cation concentration. Figure 2a compares the FD curves of torsionally constrained DNA as a function of NaCl concentration (grey-scale), along with the second derivative of the corresponding FD curves (orange-scale). Dashed lines here highlight T1–T4 in the second derivative plots (first identified in Fig. 1c). Analogous results for the case of increasing MgCl_2_ concentration are presented in Supplementary Fig. 3a. Visual inspection of Fig. 2a and Supplementary Fig. 3a reveals that ionic strength has a clear influence on the FD behaviour of torsionally constrained DNA. To provide greater insight into this, we extracted the force in the FD curves at which each transition (T1–T4) occurs (Fig. 2b–e and Supplementary Fig. 3b–e). For comparison, the DNA extension at each transition as a function of ionic strength is plotted in Supplementary Fig. 4.

The force related to T1 (Fig. 2b) changes modestly on increasing the monovalent cation concentration, indicating that NaCl has only a small influence on the switch from entropic to enthalpic stretching (Supplementary Figs 5 and 6). Meanwhile, the force at which T2 occurs increases monotonically from ∼47 pN at 0 mM NaCl, reaching a plateau (∼56 pN) at ∼500 mM NaCl (Fig. 2c). This trend is reminiscent of the change in force required to overstretch torsionally relaxed DNA under a similar salt sweep^5^,^6^. In end-closed torsionally relaxed DNA, overstretching is primarily attributed to basepair melting at ≤25 mM NaCl but is dominated by S-DNA formation at >500 mM NaCl^4^,^5^,^6^,^13^,^17^,^25^,^26^,^27^,^28^,^29^. This encourages the view that T2 is, at least in part, associated with the disruption of base pairing.

On increasing the monovalent salt concentration, the force that triggers T3 shows a clear peak at ∼400 mM NaCl (Fig. 2d). The overstretching of torsionally constrained DNA (at forces ≥110 pN) has been predicted to arise due to the formation of overwound DNA (P-DNA) in combination with underwound (for example, S-form) DNA^16^,^20^,^21^. Given this, we tentatively posit that T3 corresponds to the triple point where P-DNA can now co-exist with underwound DNA structures as well as B-DNA^20^,^21^. Since the backbones of P-DNA are tightly entwined and negatively charged^22^,^23^, high ionic strength will likely stabilize its formation through electrostatic screening. However, to form P-DNA, base pairing must be disrupted, a process that is favoured by lower ionic strengths (particularly <50 mM NaCl)^4^,^5^,^6^. This is consistent with our observation that the force at which T3 occurs shows a maximum at intermediate ionic strengths: more mechanical work is required to stabilize overwound DNA when at intermediate, rather than low or high ionic strengths (Fig. 2d; see Discussion section for more details). This in turn strengthens the view that T3 corresponds to the switch from small-scale lengthening of the DNA molecule to a relatively cooperative overstretching process that is facilitated by the formation of overwound DNA (most likely P-DNA, at least at the higher NaCl concentrations).

T4 can be considered as the point at which the entire torsionally constrained DNA molecule is overstretched; further extension leads to enthalpic stretching of the overstretched structure. As the monovalent salt concentration is increased, the force at which T4 occurs reveals a peak at ∼300 mM NaCl (Fig. 2e), indicating that a maximum in mechanical work is required to achieve T4 when exposed to such salt concentrations. We argued earlier that the overwound DNA structure (most likely P-DNA) exhibits a minimum in stability (relative to B-DNA) at ∼300–500 mM NaCl. This in turn implies a higher force for T4 at these intermediate salt concentrations. Furthermore, an increasingly flat (and thus cooperative) overstretching plateau between T3 and T4 as a function of NaCl concentration leads to an increase in the magnitude of the T4 peak in the second derivative trace (Supplementary Figs 7 and 8; Supplementary Discussion). This has the effect of further decreasing the force at which T4 occurs at high NaCl concentrations.

### Local bubble-melted DNA under applied forces of 50–110 pN

**Figure 3:**
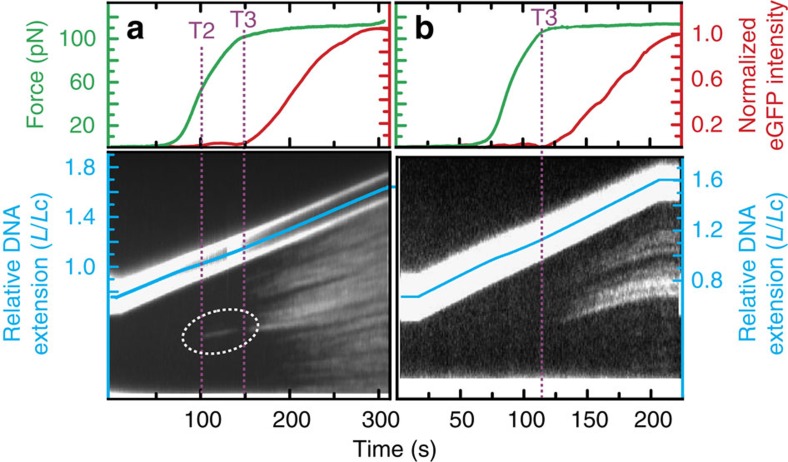
Binding of eGFP–RPA to torsionally constrained DNA. (**a**) Upper panel displays the applied force (green) and fluorescence intensity of DNA-bound eGFP–RPA (red) as torsionally constrained DNA is extended (at ∼3 μm min^−1^) in a buffer containing 10 nM eGFP–RPA. Lower panel presents the corresponding kymograph for eGFP–RPA binding to the torsionally constrained DNA molecule as a function of time (during which the molecule is being extended). The dashed white circle highlights local binding of eGFP–RPA between T2 and T3. Data here are obtained in a buffer containing 50 mM NaCl. (**b**) Analogous experiments to those reported in **a** but now with the salt concentration increased to 150 mM NaCl+20 mM MgCl_2_.

On the basis of the ionic strength dependency of T2 (Fig. 2c), we propose that local basepair melting can occur in torsionally constrained DNA between T2 and T3 (at least at lower salt concentrations). To test this hypothesis, we extended a torsionally constrained DNA molecule from 0 to 120 pN in the presence of enhanced green fluorescent protein (eGFP)-labelled replication protein A (RPA). This single-stranded DNA-binding protein has previously been used to visualize tension-induced melting of torsionally relaxed DNA^6^,^17^. Under the conditions used here, we expect to observe real-time binding of eGFP–RPA to basepair-melted domains of the torsionally constrained DNA molecule as they form^6^,^17^. The result of this measurement is displayed in Fig. 3a. Extensive eGFP–RPA binding is observed once the applied force is between that of T3 and T4, an observation that we will return to later. More strikingly, however, a small domain of RPA binding is also visualized between T2 and T3 (at forces of 50–100 pN). The RPA patch bound in this force regime was always (*N*=7) observed in the most AT-rich domain of the DNA molecule (Supplementary Fig. 9) and remains relatively constant in length until the applied force approaches that of T3.

We repeated these measurements at a higher ionic strength (150 mM NaCl+20 mM MgCl_2_). Under these conditions (Fig. 3b) no eGFP–RPA is bound to torsionally constrained DNA at forces lower than that of T3. There is, however, no significant difference in the binding affinity of RPA for basepair-melted DNA between 50 mM NaCl and 150 mM NaCl+20 mM MgCl_2_ (refs 6, 30). In agreement with our analysis of Fig. 2c, the above findings indicate that torsionally constrained DNA (in at least ≤50 mM NaCl) has a propensity to develop localized basepair-melted domains (in AT-rich sequences) when the molecule is between T2 and T3. Close inspection of Fig. 3a also indicates that the eGFP–RPA bound to basepair-melted DNA between T2 and T3 unbinds immediately before T3. Since T3 is associated with the triple point where overwound DNA (likely P-DNA) co-exists with underwound DNA and B-DNA^20^,^21^, this reproducible observation (*N*∼5) may indicate that P-DNA also occurs preferentially in AT-rich domains of DNA; in which case P-DNA can only form by dissociating the RPA from the locally melted DNA.

### P-DNA, bubble-melted DNA and S-DNA at forces ≥∼110 pN

It has been predicted^16^,^20^,^21^ that when the force applied to torsionally constrained DNA is ≥110 pN (between T3 and T4), 80% of the overstretched state exists in an underwound configuration, while 20% adopts an overwound (namely P-DNA) form. In contrast, torsionally relaxed DNA is composed of only underwound DNA structures when overstretched. We set out to test this interpretation, by measuring the fraction of overstretched DNA that is underwound in each case. Underwound overstretched DNA is predominantly basepair-melted at low ionic strengths (≤25 mM NaCl) and progressively base-paired (in the form of S-DNA) as the ionic strength is increased^4^,^5^,^6^. Since there is no known probe for S-DNA, we choose to use the binding of eGFP–RPA as a signal for basepair-melted DNA. Note, however, that the relative ratio of bubble-melted DNA to S-DNA at a given salt concentration is not expected to be affected by the presence or absence of torsional constraint. In this way, we use eGFP–RPA fluorescence to detect the relative change in the fraction of underwound DNA between torsionally relaxed DNA and torsionally constrained DNA.

**Figure 4:**
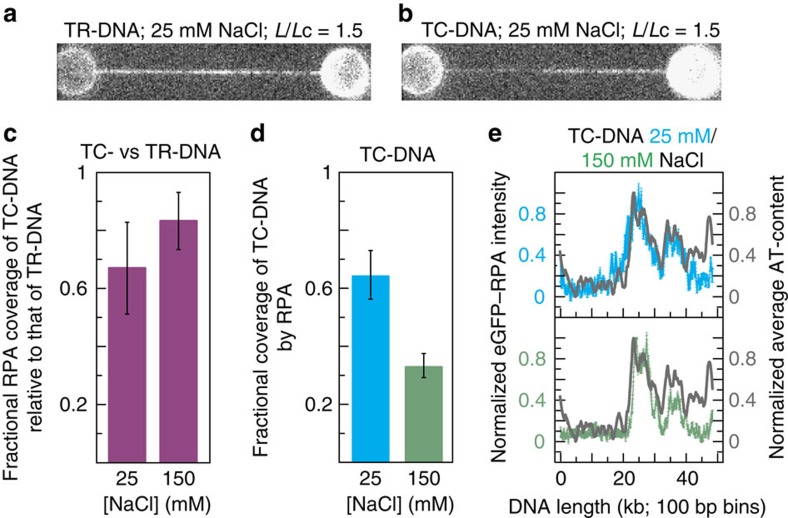
DNA coverage by RPA decreases when under torsional constraint and at elevated ionic strengths. (**a**,**b**) Sample fluorescence images of eGFP–RPA bound to torsionally relaxed (TR-)DNA and torsionally constrained (TC-)DNA, respectively. Data obtained at *L*/*L*c=1.5 and in 25 mM NaCl. (**c**) Fractional eGFP–RPA coverage of torsionally constrained (TC-)DNA relative to that of torsionally relaxed (TR-)DNA, shown for both 25 mM NaCl and 150 mM NaCl. For each salt condition, three comparisons were made (*N*=3), consisting of DNA molecules at an identical *L*/*L*c (1.2≤*L*/*L*c≥1.6). (**d**) Fractional coverage of torsionally constrained (TC-)DNA by eGFP–RPA at *L*/*L*c=1.2 in 25 mM NaCl (blue) and 150 mM NaCl (green). *N*=3 in each case. (**e**) eGFP–RPA fluorescence intensity along the length of the torsionally constrained (TC-)DNA molecule (averaged over *N*=6, with error bars shown) in 25 mM NaCl (blue) and 150 mM NaCl (green). Fluorescence intensity profiles are oriented in the direction that best matches the average AT-content of the DNA molecule (grey). Errors are s.e.m.

Figure 4a,b compares sample fluorescence images recorded for eGFP–RPA bound to torsionally relaxed DNA and torsionally constrained DNA, respectively, at *L*/*L*c=1.5 and in 25 mM NaCl. From these images it can be observed that the fraction of the DNA molecule covered by eGFP–RPA is lower in the case of torsionally constrained DNA than in torsionally relaxed DNA. Figure 4c demonstrates that this is a consistent observation: the coverage of torsionally constrained DNA by eGFP–RPA is lower than that of torsionally relaxed DNA by 33±16% in 25 mM NaCl and 17±10% in 150 mM NaCl (errors are s.e.m.). Such changes in RPA coverage would not be expected if the protein could bind to P-DNA: in that event, RPA would bind to a similar fraction of the torsionally constrained DNA molecule as it would for torsionally relaxed DNA. On the basis of this observation, we conclude that RPA is unlikely to bind to P-DNA under our experimental conditions.

From Fig. 4a,b, and assuming that the average size of a basepair-melted bubble is similar in both torsionally relaxed and torsionally constrained DNA, our results indicate that the fraction of the overstretched state (between T3 and T4) that is bubble-melted is lower in torsionally constrained DNA than in torsionally relaxed DNA. Furthermore, bubble-melted DNA is expected to exhibit an underwound structure during overstretching^5^,^6^. With this in mind, and based on the relative changes in RPA coverage shown in Fig. 4c, we can determine that between T3 and T4 the percentage of overstretched torsionally constrained DNA that is underwound is 67±16% in 25 mM NaCl and 83±10% in 150 mM NaCl (errors are s.e.m.). This indicates that overwound DNA (most likely P-DNA) accounts for ∼20–30% of the overstretched state of torsionally constrained DNA at forces >∼110 pN, in excellent agreement with the prediction by Léger *et al*.^16^

We also note that between T3 and T4 the total coverage of torsionally constrained DNA by eGFP–RPA decreases from 65±9% to 34±4% (at *L*/*L*c=1.2) on increasing the NaCl concentration from 25 to 150 mM (Fig. 4d; errors are s.e.m.). This quantifies a trend that was observed qualitatively in Fig. 3a,b. Since the binding affinity of RPA is invariant over this range of salt concentrations^6^,^30^, we conclude that, as the ionic strength increases, regions of bubble-melted DNA are replaced by (base-paired) S-DNA, mirroring a similar trend in torsionally relaxed DNA^4^,^5^,^6^. As also expected from studies of torsionally relaxed DNA^6^, those domains of torsionally constrained DNA that remain bubble-melted (identified by the binding of eGFP–RPA) show a strong preference for AT-rich sequences (Fig. 4e).

## Discussion

We provide compelling new evidence that torsionally constrained DNA displays at least two structural transitions during the overstretching process. At forces >∼110 pN (T3), an overwound DNA configuration (structurally distinct from that of B-DNA) co-exists with underwound DNA structures (bubble-melted DNA/S-DNA) in a ∼20:80 ratio. Our findings reveal that the energetic stability of such overwound DNA shows a marked salt-dependence, whereby it is stabilized by lower (<200 mM NaCl) and higher (>600 mM NaCl) regimes of ionic strength. On the basis of this behaviour, we attribute the structure of overwound DNA created in this force range to that of P-DNA (at least for NaCl concentrations >∼50 mM), strongly supporting earlier theoretical predictions^16^,^20^,^21^. As a caveat, we raise the possibility that at low salt concentrations (<∼50 mM NaCl), it is possible that the limited electrostatic shielding may disfavour the formation of P-DNA owing to the close juxtaposition of the tightly entwined backbones. In this case, the overwound DNA might be expected to exhibit a structure more akin to overwound bubble-melted DNA.

At lower forces (between T2 and T3: ∼50–110 pN), we reveal that localized basepair melting can occur (at least for ≤50 mM NaCl). Since changes in FD behaviour indicate that P-DNA is not viable between T2 and T3 (Fig. 2), this raises an intriguing and important question: how is the base-pairing disrupted while Lk is kept constant in this force range?

**Figure 5:**
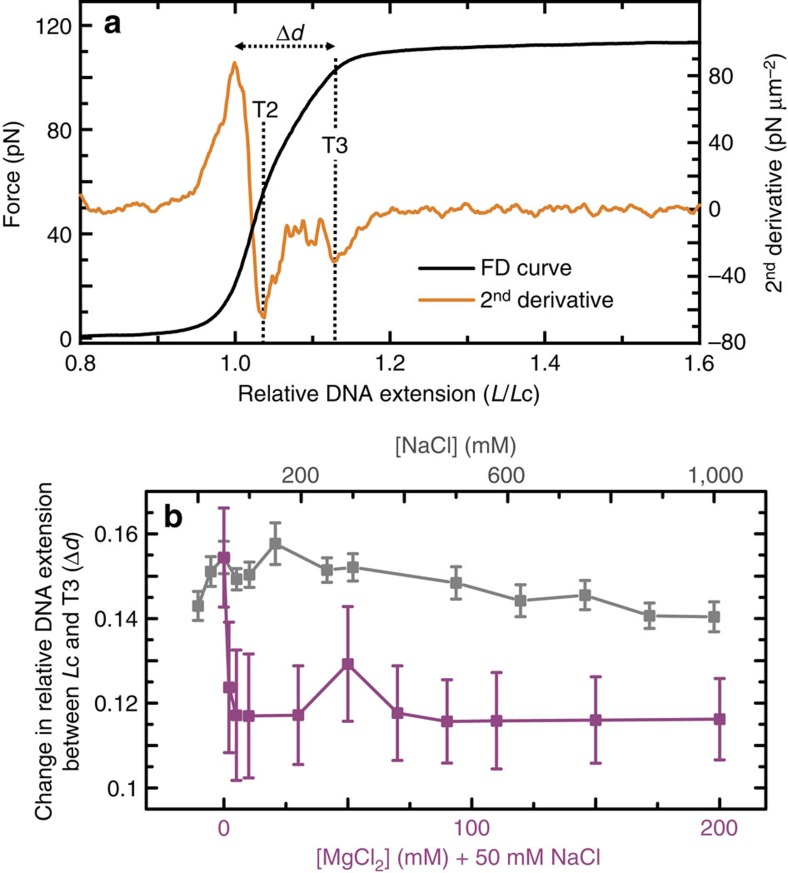
Localized overstretching occurs in torsionally constrained DNA between T2 and T3. (**a**) Average FD curve of torsionally constrained DNA in 200 mM MgCl_2_+50 mM NaCl (black) along with the corresponding second derivative profile (orange). The distance (Δ*d*) between the relative DNA extension at *L*c and that at T3 is highlighted. (**b**) Plot showing how the distance Δ*d* varies as a function of both NaCl (grey) and MgCl_2_ (purple) concentration. Errors are s.e.m.

One possibility is that, at forces higher than T2, the two DNA backbones are squeezed together latterly while basepairs are flipped outwards. This mechanism would not require any change to Lk and could induce a small elongation of the molecule until P-DNA is formed at T3. Basepair melting is either prohibited or significantly suppressed at the highest ionic strengths investigated here (1 M NaCl or 200 mM MgCl_2_). Nevertheless, as Fig. 5 reveals, the change in relative extension of torsionally constrained DNA from *L*c until T3 (Δ*d*) tends to a lower value of ∼11.8% as the ionic strength is increased. This, still significant extension, implies that a ‘latterly squeezed' basepair-melted structure cannot completely explain the observed force-extension behaviour, at least at high salt concentrations.

The force range between T2 and T3 coincides with where torsionally relaxed DNA overstretches. Given this, we posit that locally overstretched domains (bubble-melted at low salt; S-DNA at high salt) likely occur in torsionally constrained DNA in this force range. This finding agrees with and builds on two theoretical predictions, which had thus far remained untested^20^,^21^. We, therefore, conclude that the experimentally observed occurrence of basepair melting between T2 and T3 (Figs 2c and 3a) is due to the formation of locally melted underwound domains. As the ionic strength increases, these underwound regions will largely exist as S-DNA. It is worth noting that this can also explain the observed increase in steepness of the FD curves between T2 and T3 as the ionic strength increases: bubble-melted DNA is expected to exhibit a greater extensibility than S-DNA (where base-pairing will make the molecule stiffer).

To maintain a constant Lk, however, such local helical unwinding must be accompanied by sufficient small-scale overwinding in other domains of the double-helix. In the range of T2–T3 (∼50–110 pN), we cannot invoke P-DNA as the accompanying overwound structure, as explained above; thus, we suggest that overwound domains in this force regime take the form of B-DNA with a slightly greater helicity.

**Figure 6:**
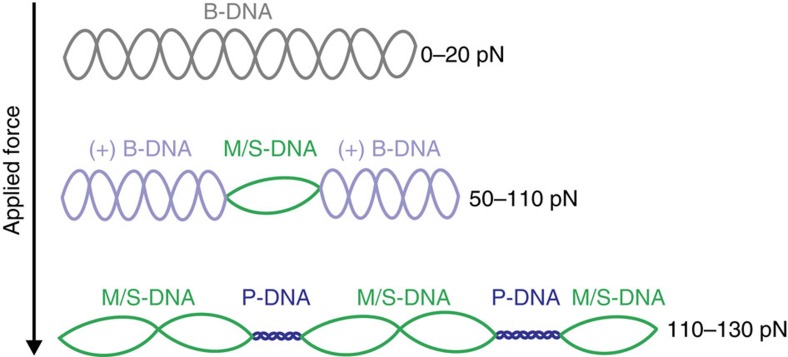
Model for structural rearrangement of torsionally constrained DNA under tension. At forces of ∼50–110 pN, the applied strain is sufficient to induce local regions of the molecule to form non-B-form underwound structures, namely bubble-melted (M) DNA and S-DNA at lower and higher ionic strengths, respectively. This is absorbed by the rest of the duplex which forms slightly overwound (+) B-DNA. At yet higher forces (∼110–130 pN) P-DNA becomes a viable overwound structure, enabling the rest of the molecule to form underwound structures (M/S-DNA). Note that, for simplicity, base-pairing is not shown in this schematic.

Taken together, our findings highlight that DNA under torsional constraint displays a rich and variable heterogeneous plasticity as applied tension is increased. This is summarized schematically in Fig. 6. We note that our experiments are performed on DNA with Lk fixed to that of B-DNA; the relative fraction of underwound and overwound DNA generated through applied tension is expected to change depending on the initial Lk (that is, the degree of supercoiling)^16^,^20^,^21^. While cellular DNA is rarely thought to encounter some of the higher tensions investigated in this work, it is regularly under torsional constraint. We, therefore, suggest that the observed structural rearrangements may enable genomic DNA to absorb local changes in Lk created by the action of proteins. We have also presented a novel analytical approach for using the second derivatives of FD curves to extract highly quantitative information on force-induced structural transitions of DNA. In this way, the current work is expected to pave the way for a new level of theory capable of both modelling and predicting the mechanical properties of DNA. More generally, however, we propose that the above analytical approach could also gain traction as a means to study how DNA-binding proteins influence the mechanics of DNA.

## Methods

### Buffer conditions

All data were obtained in a buffer containing 20 mM tris pH 7.6 at room temperature (21±2 °C). Note that magnesium concentration sweeps were performed in a background of 50 mM NaCl.

### DNA construct preparation

End-capped DNA molecules (based on λ-DNA, ∼48.5 kb) were prepared with and without torsional constraint when bound to optically trapped beads. End-closed DNA was generated by ligating an end-cap to each terminal *cos*-site of λ-DNA. Each end-cap (labelled with four biotins) was composed of a 5 T loop adjoined to a 12-bp double-stranded stem and a 12 nucleotide single-stranded overhang (Supplementary Figs 1 and 2). The constructs differed only in the number of biotin moieties connected to each streptavidin-coated and optically trapped bead. In the torsionally relaxed case, each end of the DNA molecule was tethered to a bead, but at least one of the ends formed only a single biotin-streptavidin link. In this way, the DNA molecule could rotate around the sole biotin-strepatavidin bond when under an applied tension^5^,^6^,^18^. Importantly, this construct had no free ends or nicks in the backbone and, as a result, strand peeling during overstretching was prohibited^5^,^6^,^18^. In the torsionally constrained design, each end of the DNA molecule was tethered to a streptavidin-coated and optically trapped bead with between two and four biotin moieties. This configuration prohibited rotation of the DNA molecule with respect to the beads. Moreover, since the diameter of the DNA molecule (2 nm) is orders of magnitude smaller than that of the optically trapped beads (4.5 μm), any torque generated by stretching the DNA molecule was insufficient to cause rotation of the beads themselves^17^. These two restrictions rendered this type of DNA molecule torsionally constrained. See Supplementary Methods for more details.

### Optical trapping and fluorescence microscopy

Experimental data were obtained using a combined fluorescence and dual-beam optical trapping instrument, described in detail elsewhere^31^,^32^. All second derivatives of FD curves were obtained using a Savitzky-Golay (20 point) smoothing filter.

### RPA as a structural probe

The fluorescent labelling of eGFP–RPA has been described previously^33^, while a full characterization of RPA binding to overstretched DNA is documented in (ref. 6). Provided the RPA concentration is sufficiently low (≤10 nM), any perturbation to the DNA structure induced by RPA binding is minimal^6^. In all experiments reported in this article, the RPA concentration was 10 nM and the incubation time for the DNA in RPA was 20 s, unless stated otherwise.

### Data availability

The data that support the findings of this study are either available within the article (and the Supplementary Information) or are available from the corresponding authors on request.

## Additional information

**How to cite this article:** King, G. A. *et al*. Unravelling the structural plasticity of stretched DNA under torsional constraint. *Nat. Commun.* 7:11810 doi: 10.1038/ncomms11810 (2016).

## Supplementary Material

Supplementary Information Supplementary Figures 1-9, Supplementary Discussion, Supplementary Methods and Supplementary References

## References

[b1] Milstein J. N. & Meiners J. C. On the role of DNA biomechanics in the regulation of gene expression. J. R. Soc. Interface 8, 1673–1681 (2011). 21865249 10.1098/rsif.2011.0371 PMC3203490

[b2] Rief M., Clausen-Schaumann H. & Gaub H. E. Sequence-dependent mechanics of single DNA molecules. Nat. Struct. Biol. 6, 346–349 (1999). 10201403 10.1038/7582

[b3] Sheinin M. Y., Forth S., Marko J. F. & Wang M. D. Underwound DNA under tension: structure, elasticity, and sequence-dependent behaviors. Phys. Rev. Lett. 107, 108102–108106 (2011). 21981534 10.1103/PhysRevLett.107.108102 PMC3201814

[b4] Bosaeus N. . Tension induces a base-paired overstretched DNA conformation. Proc. Natl Acad. Sci. USA 109, 15179–15184 (2012). 22949705 10.1073/pnas.1213172109 PMC3458322

[b5] Zhang X. . Revealing the competition between peeled ssDNA, melting bubbles, and S-DNA during DNA overstretching by single-molecule calorimetry. Proc. Natl Acad. Sci. USA 110, 3865–3870 (2013). 23431154 10.1073/pnas.1213740110 PMC3593865

[b6] King G. A. . Revealing the competition between peeled ssDNA, melting bubbles, and S-DNA during DNA overstretching using fluorescence microscopy. Proc. Natl Acad. Sci. USA 110, 3859–3864 (2013). 23431161 10.1073/pnas.1213676110 PMC3593895

[b7] Forth S., Sheinin M. Y., Inman J. & Wang M. D. Torque measurement at the single-molecule level. Annu. Rev. Biophys. 42, 583–604 (2013). 23541162 10.1146/annurev-biophys-083012-130412 PMC5515228

[b8] Meng H., Bosman J., van der Heijden T. & van Noort J. Coexistence of twisted, plectonemic, and melted DNA in small topological domains. Biophys. J. 106, 1174–1181 (2014). 24606941 10.1016/j.bpj.2014.01.017 PMC4026787

[b9] Heller I., Hoekstra T. P., King G. A., Peterman E. J. G. & Wuite G. J. L. Optical tweezers analysis of DNA–protein complexes. Chem. Rev. 114, 3087–3119 (2014). 24443844 10.1021/cr4003006

[b10] Vlijm R., Mashaghi A., Bernard S., Modesti M. & Dekker C. Experimental phase diagram of negatively supercoiled DNA measured by magnetic tweezers and fluorescence. Nanoscale 7, 3205–3216 (2015). 25615283 10.1039/c4nr04332d

[b11] Sitters G. . Acoustic force spectroscopy. Nat. Methods 12, 47–50 (2015). 25419961 10.1038/nmeth.3183

[b12] Gore J. . DNA overwinds when stretched. Nature 442, 836–839 (2006). 16862122 10.1038/nature04974

[b13] Gross P. . Quantifying how DNA stretches, melts and changes twist under tension. Nat. Phys. 7, 731–736 (2011).

[b14] Cluzel P. . DNA: an extensible molecule. Science 271, 792–794 (1996). 8628993 10.1126/science.271.5250.792

[b15] Smith S. B., Cui Y. & Bustamante C. Overstretching B-DNA: the elastic response of individual double-stranded and single-stranded DNA molecules. Science 271, 795–799 (1996). 8628994 10.1126/science.271.5250.795

[b16] Léger J. F. . Structural transitions of a twisted and stretched DNA molecule. Phys. Rev. Lett. 83, 1066–1069 (1999).

[b17] van Mameren J. . Unraveling the structure of DNA during overstretching by using multicolor, single-molecule fluorescence imaging. Proc. Natl Acad. Sci. USA 106, 18231–18236 (2009). 19841258 10.1073/pnas.0904322106 PMC2775282

[b18] Paik D. H. & Perkins T. T. Overstretching DNA at 65 pN does not require peeling from free ends or nicks. J. Am. Chem. Soc. 133, 3219–3221 (2011). 21207940 10.1021/ja108952v

[b19] Strzelecki J., Peplowski L., Lenartowski R., Nowak W. & Balter A. Mechanical transition in a highly stretched and torsionally constrained DNA. Phys. Rev. E 89, 020701–020705 (2014). 10.1103/PhysRevE.89.020701 25353406

[b20] Sarkar A., Léger J. F., Chatenay D. & Marko J. F. Structural transitions in DNA driven by external force and torque. Phys. Rev. E 63, 051903–051912 (2001). 10.1103/PhysRevE.63.051903 11414929

[b21] Marko J. F. & Neukirch S. Global force-torque phase diagram for the DNA double-helix: structural transitions, triple points, and collapsed plectonemes. Phys. Rev. E 88, 062722–062739 (2013). 10.1103/PhysRevE.88.062722 PMC3936674 24483501

[b22] Allemand J. F., Bensimon D., Lavery R. & Croquette V. Stretched and overwound DNA forms a pauling-like structure with exposed bases. Proc. Natl Acad. Sci. USA 95, 14152–14157 (1998). 9826669 10.1073/pnas.95.24.14152 PMC24342

[b23] Wereszczynski J. & Andricioaei I. On structural transitions, thermodynamic equilibrium, and the phase diagram of DNA and RNA duplexes under torque and tension. Proc. Natl Acad. Sci. USA 103, 16200–16205 (2006). 17060631 10.1073/pnas.0603850103 PMC1637560

[b24] Schlick T., Li B. & Olson W. K. The influence of salt on the structure and energetics of supercoiled DNA. Biophys. J. 67, 2146–2166 (1994). 7696459 10.1016/S0006-3495(94)80732-5 PMC1225601

[b25] Cocco S., Yan J., Léger J. F., Chatenay D. & Marko J. F. Overstretching and force-driven strand separation of double-helix DNA. Phys. Rev. E 70, 011910 (2004). 10.1103/PhysRevE.70.011910 15324091

[b26] Fu H., Chen H., Marko J. F. & Yan J. Two distinct overstretched DNA states. Nucleic Acids Res. 38, 5594–5600 (2010). 20435680 10.1093/nar/gkq309 PMC2938222

[b27] Fu H. . Transition dynamics and selection of the distinct S-DNA and strand unpeeling modes of double helix overstretching. Nucleic Acids Res. 39, 3473–3481 (2011). 21177651 10.1093/nar/gkq1278 PMC3082884

[b28] Zhang X., Chen H., Fu H., Doyle P. S. & Yan J. Two distinct overstretched DNA structures revealed by single-molecule thermodynamics measurements. Proc. Natl Acad. Sci. USA 109, 8103–8108 (2012). 22532662 10.1073/pnas.1109824109 PMC3361402

[b29] Bongini L., Melli L., Lombardi V. & Bianco P. Transient kinetics measured with force steps discriminate between double-stranded DNA elongation and melting and define the reaction energetics. Nucleic Acids Res. 42, 3436–3449 (2014). 24353317 10.1093/nar/gkt1297 PMC3950695

[b30] De Vlaminck I. . Torsional regulation of hRPA-induced unwinding of double-stranded DNA. Nucleic Acids Res. 38, 4133–4142 (2010). 20197317 10.1093/nar/gkq067 PMC2896508

[b31] van Mameren J. . Counting RAD51 proteins disassembling from nucleoprotein filaments under tension. Nature 457, 745–748 (2009). 19060884 10.1038/nature07581 PMC3871861

[b32] Gross P., Farge G., Peterman E. J. G. & Wuite G. J. L. Combining optical tweezers, single-molecule fluorescence microscopy, and microfluidics for studies of DNA-protein interactions. Methods Enzymol. 475, 427–453 (2010). 20627167 10.1016/S0076-6879(10)75017-5

[b33] Modesti M. Fluorescent labeling of proteins. Methods Mol. Biol. 783, 101–120 (2011). 21909885 10.1007/978-1-61779-282-3_6

