# Supplementary material for: Unravelling the structural plasticity of stretched DNA under torsional constraint
Source: Nat Commun. 2016 Jun 6;7:11810. doi: 10.1038/ncomms11810 (PMC4897764; doi:10.1038/ncomms11810)
Supplement: Supplementary Information — Supplementary Figures 1-9, Supplementary Discussion, Supplementary Methods and Supplementary References [file ncomms11810-s1.pdf]

Supplementary Figures

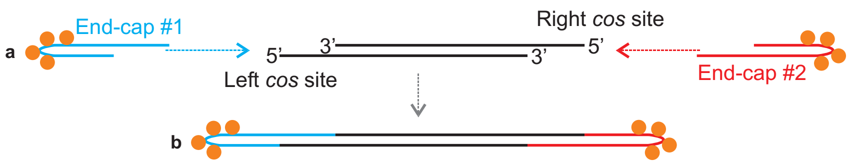

Supplementary Figure 1: Formation of end-closed DNA constructs. a, Two end caps (#1 and #2, each containing four biotin moieties (depicted in orange)) are ligated to the left and right *cos* sites of -DNA, respectively. **b**, Provided the DNA molecule contains no nicks in the backbones, this results in a topologically-closed DNA molecule.

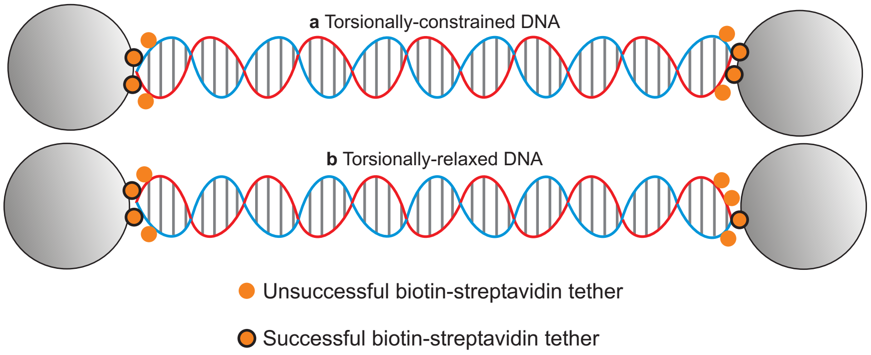

Supplementary Figure 2: Formation of end-closed torsionally-constrained DNA and end-closed torsionally-relaxed DNA. a, Torsionally-constrained DNA is generated in cases where end closed DNA is successfully tethered to streptavidin-coated beads (grey circles) via at least two biotin moieties (orange circles) on each end of the molecule. **b**, Torsionally-relaxed DNA results when only a single biotin moiety is successfully tethered to one or both of the two streptavidin-coated beads.

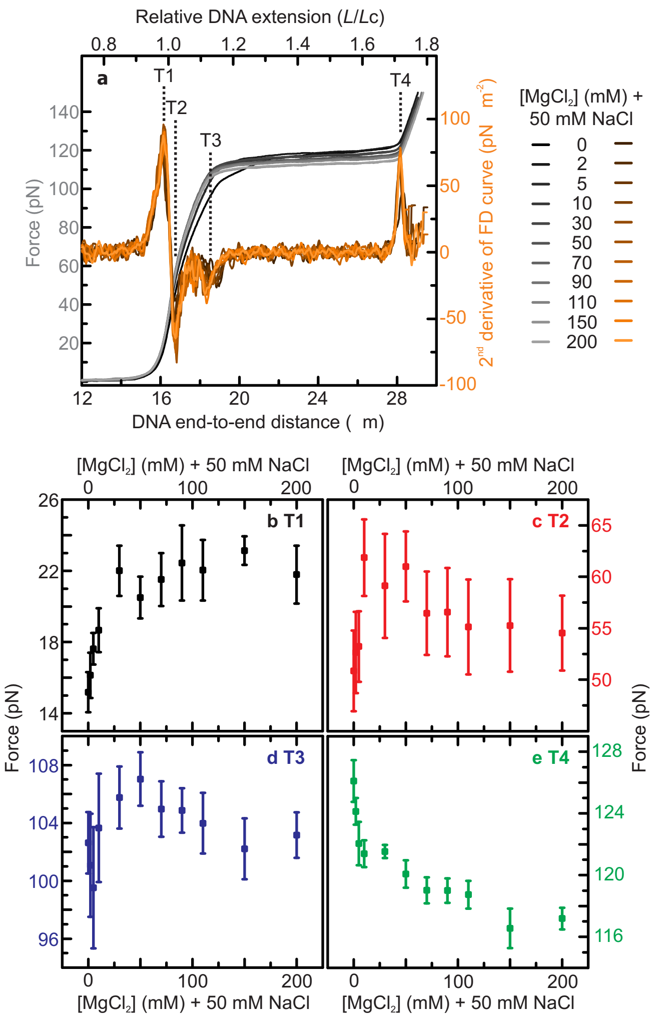

Supplementary Figure 3: Effect of divalent salt on the FD curves for torsionally-constrained DNA. a, Average FD curves (grey-scale), together with the 2nd derivative of each curve (orange-scale) as the concentration of $MgCl_2$ is increased. **b e**, Force associated with torsionally-constrained DNA at T1 T4, respectively, as a function of $MgCl_2$ concentration. All data were recorded in a buffer of 20 mM tris pH 7.6. Errors are standard errors of the mean (SEM) (N~10).

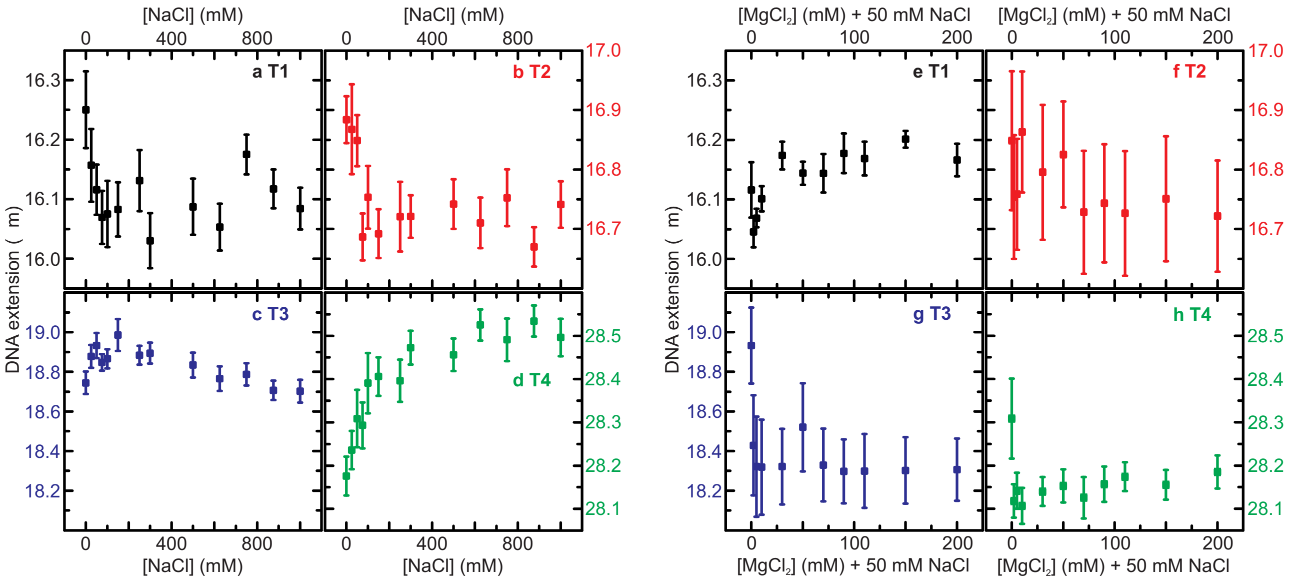

Supplementary Figure 4: DNA extension at T1 T4 as a function of ionic strength. a d, DNA extension at T1 T4 as a function of NaCl concentration. **e h**, DNA extension at T1 T4 as a function of $MgCl_2$ concentration. The buffer used was 20 mM tris pH 7.6. Errors are SEM (N~10).

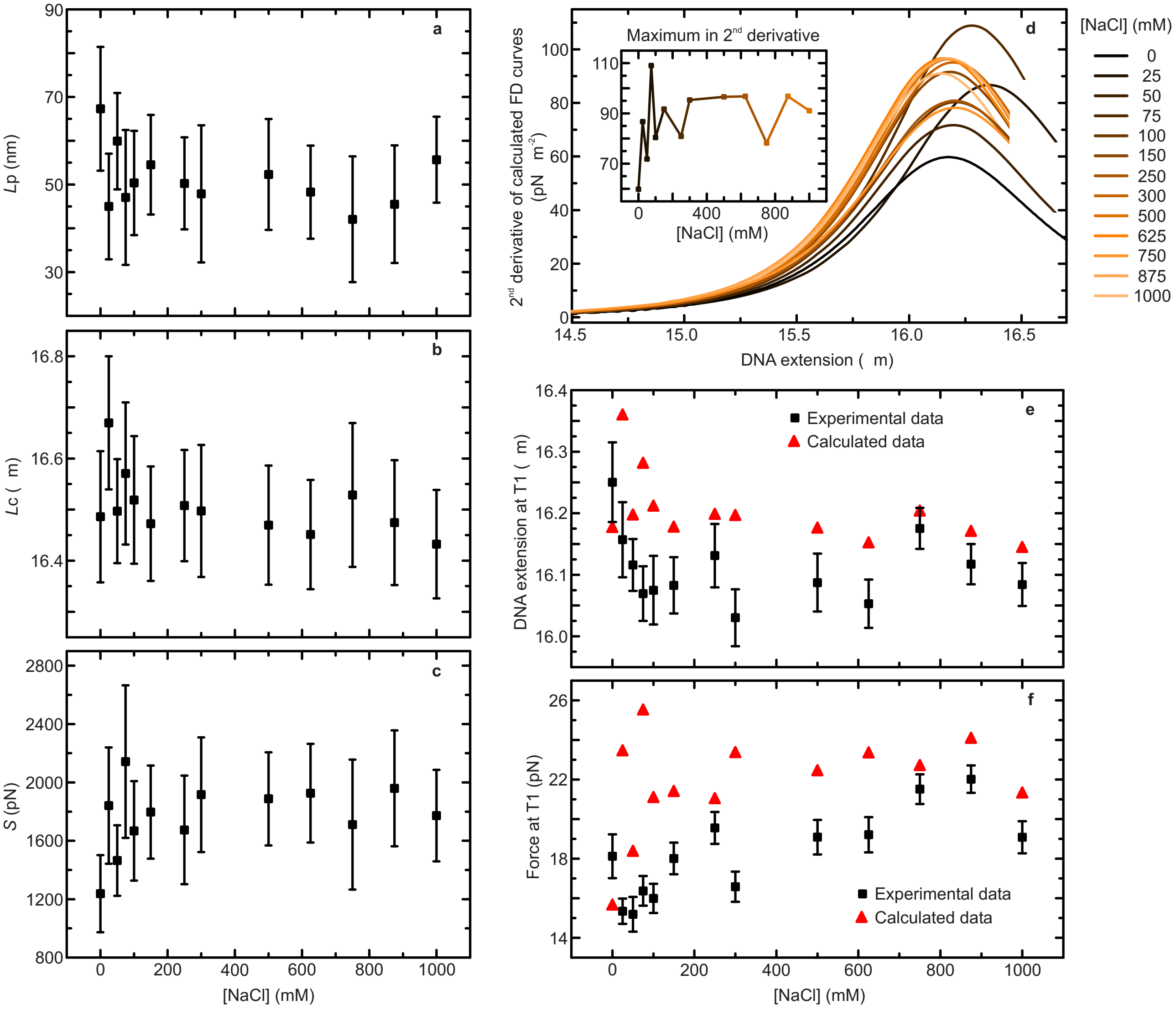

Supplementary Figure 5: The force and extension at which T1 occurs as a function of NaCl concentration is determined primarily by the values of *L*p and *S* for the DNA molecule. a c, Results of fits of the eWLC model to experimentally derived FD curves of torsionally-constrained DNA (up to 30 pN) as a function of NaCl concentration. **d**, 2nd derivative of calculated FD curves (created using the fitted *L*p, *L*c and *S* values in panels a c) as a function of ionic strength. Note that the 2nd derivative is derived using a Savitzky-Golay (20 point) smoothing filter. Inset highlights the maximum in the 2nd derivative peak as a function of NaCl concentration. **e**, DNA extension in the calculated FD curves at T1 as a function of NaCl concentration. **f**, Force in the calculated FD curves at T1 as a function of NaCl concentration. The buffer used was 20 mM tris pH 7.6. Errors are SEM (N~10). See Supplementary Discussion for more details.

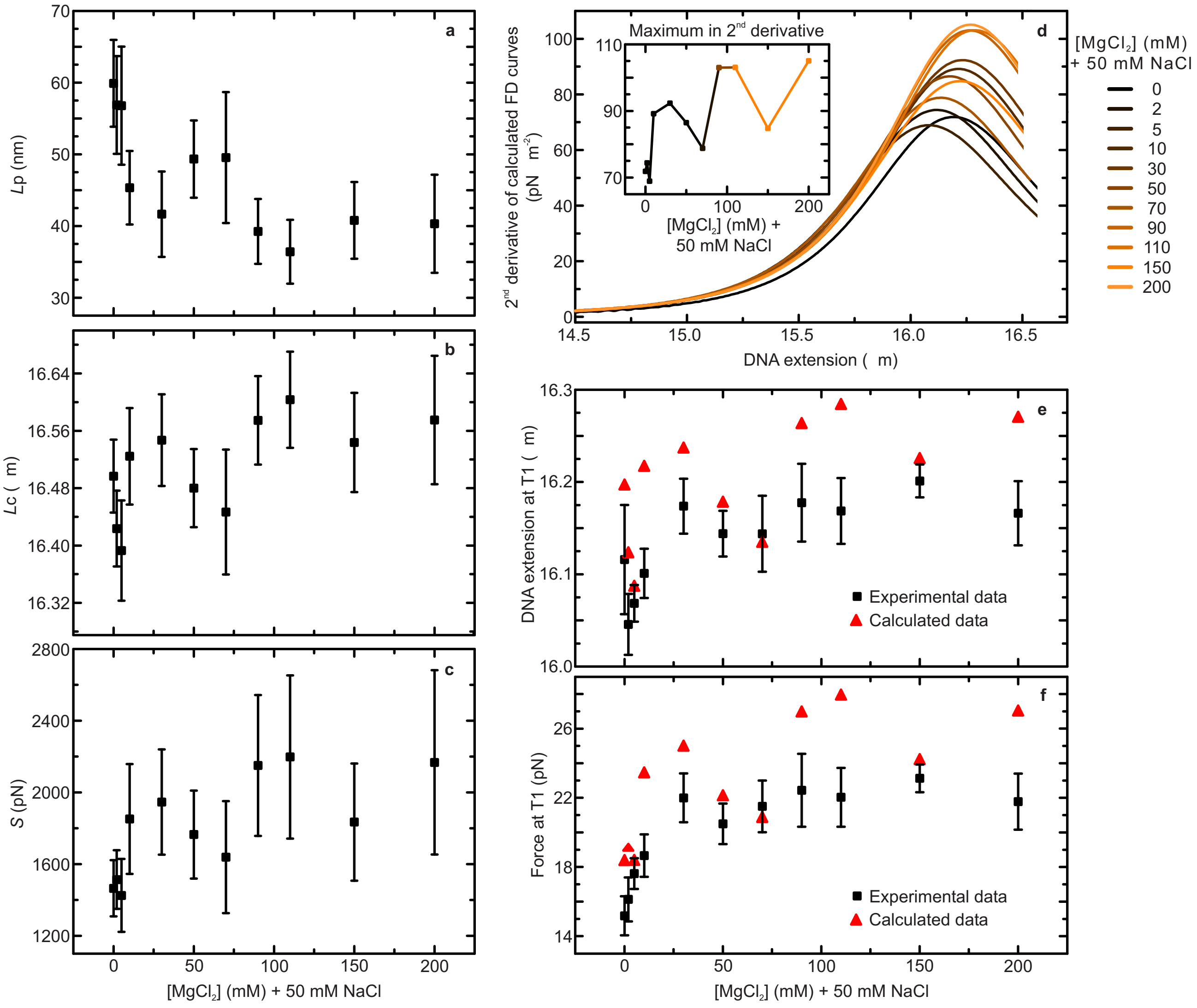

Supplementary Figure 6: The force and extension at which T1 occurs as a function of MgCl$_2$ concentration is determined primarily by the values of *L*p and *S* for the DNA molecule. a c, Results of fits of the eWLC model to experimentally derived FD curves of torsionally-constrained DNA (up to 30 pN) as a function of MgCl$_2$ concentration. **d**, 2nd derivative of calculated FD curves (created using the fitted *L*p, *L*c and *S* values in panels a c) as a function of ionic strength. Note that the 2nd derivative is derived using a Savitzky-Golay (20 point) smoothing filter. Inset highlights the maximum in the 2nd derivative peak as a function of MgCl$_2$ concentration. **e**, DNA extension in the calculated FD curves at T1 as a function of MgCl$_2$ concentration. **f**, Force in the calculated FD curves at T1 as a function of MgCl$_2$ concentration. The buffer used was 20 mM tris pH 7.6. Errors are SEM (N~10). See Supplementary Discussion for more details.

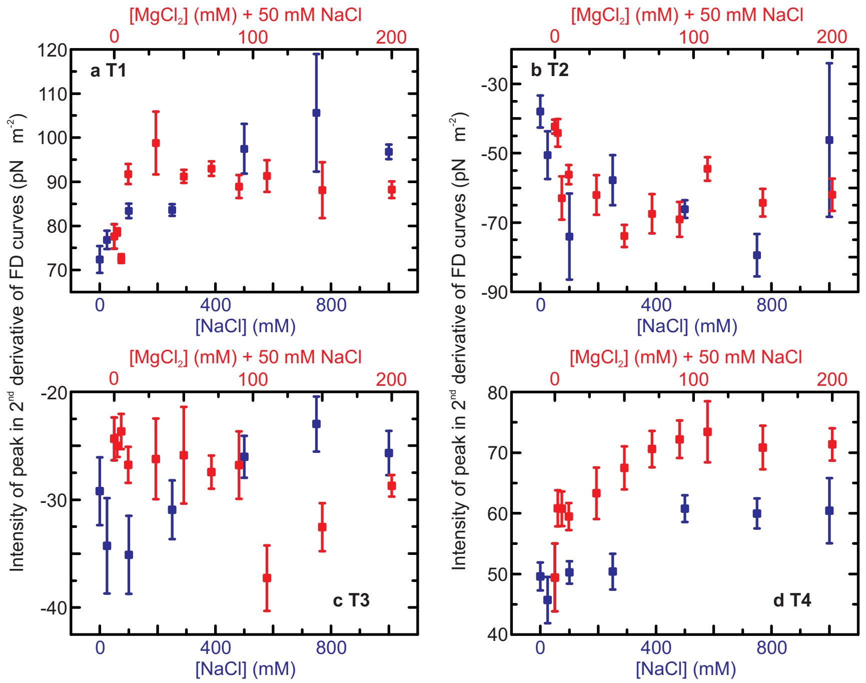

Supplementary Figure 7: Peak intensities of T1 T4 in the 2nd derivative profiles shown in Fig. 2a and Supplementary Fig. 3a as a function of ionic strength. Peak intensities are shown for T1 (**a**), T2 (**b**), T3 (**c**) and T4 (**d**) as a function of both NaCl (blue) and $MgCl_2$ (red) concentration. The buffer used was 20 mM tris pH 7.6. Errors are SEM (N~10).

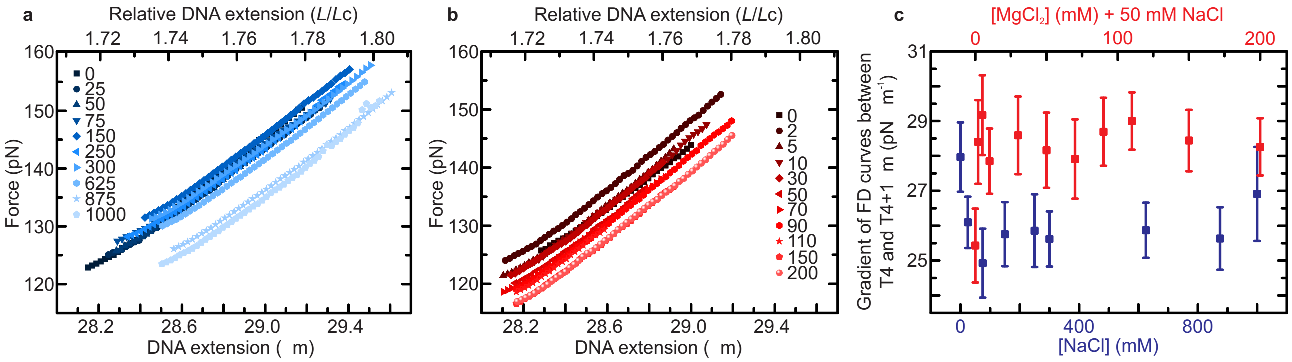

Supplementary Figure 8: Changes in NaCl and $MgCl_2$ concentration, respectively, have generally only a small influence on the stiffness of torsionally-constrained DNA at forces > T4. **a**, Average FD curves for torsionally-constrained DNA molecules between T4 and T4 + 1 m as a function of NaCl concentration (highlighted with blue-scale symbols). **b**, Average FD curves for torsionally-constrained DNA molecules between T4 and T4 + 1 m as a function of $MgCl_2$ concentration + 50 mM NaCl (highlighted with red-scale symbols). **c**, Average gradient (linear fit) of FD curves for torsionally-constrained DNA molecules between T4 and T4 + 1 m as a function of both NaCl (dark blue) and $MgCl_2$ (red) concentration. Note, however, that magnesium stiffens torsionally-constrained DNA more than NaCl at forces > T4. All data were obtained in a buffer of 20 mM tris pH 7.6. Errors are SEM (N~10).

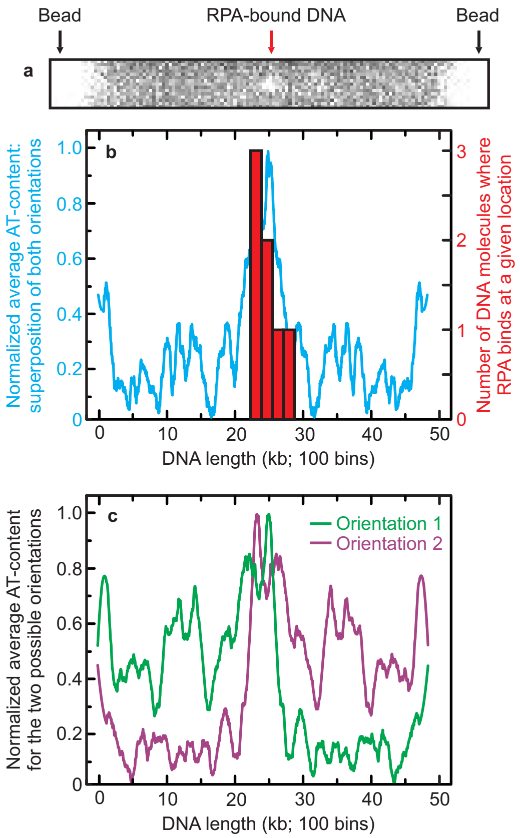

Supplementary Figure 9: RPA binds to the most AT-rich domain of torsionally-constrained DNA when between T2 and T3. a, Typical fluorescence image recorded for torsionally-constrained DNA between T2 and T3. The trapped beads are observed on either end, while a small accumulation of eGFP-RPA is seen in roughly the middle of the DNA molecule. **b**, The red histogram shows the number of torsionally-constrained DNA molecules between T2 and T3 where eGFP-RPA is visualized at a specific DNA location. Here, we assume that a signal greater than 2 standard deviations above the background signal corresponds to eGFP-RPA binding. The blue trace displays a superposition of the average AT-content of lambda DNA for both possible orientations. Note that in Fig. 4e there was a clear match between the distinctive eGFP-RPA binding pattern and the average AT-content of the DNA molecule; this in turn allowed us to determine the orientation of the DNA molecule. In the current case, however, no such match can easily be made (due to the low coverage). Therefore, in panel b we present a superposition of the two possible orientations of the DNA molecule. Nonetheless, this is still sufficient to determine that, between T2 and T3, RPA binds only to a small location of the DNA molecule (centered at ~25 kb) that has the highest AT-content. **c**, Overlay of the average AT-content of lambda DNA for both possible orientations. The superposition of these two profiles yields the blue trace in panel b.

Supplementary Discussion

Forces associated with T1–T4 as a function of divalent salt ($MgCl_2$) concentration. Building upon our investigation into the effects of monovalent salt (NaCl) on the FD curves of torsionally-constrained DNA (Fig. 2), Supplementary Fig. 3 highlights how the forces associated with T1–T4 vary as a function of divalent salt ($MgCl_2$) concentration. As the $MgCl_2$ concentration is increased, the trends in FD behaviour of torsionally-constrained DNA are generally similar to those observed during the NaCl concentration sweep (Fig. 2). Interestingly, however, the effects of $MgCl_2$ are typically ~100-fold higher than for NaCl. This is in accordance with previous studies that indicate that ~100-fold higher sodium concentration is required to induce the same effective DNA diameter as magnesium does (1).

Ionic-strength dependency of T1. Panels a–c of Supplementary Fig. 5 report the changes in persistence length (*L*p), contour length (*L*c) and stretch modulus (*S*), respectively, of torsionally-constrained DNA as a function of NaCl concentration. These were determined here by fitting FD curves of torsionally-constrained DNA (up to 30 pN) to the extensible (e)WLC model. This reveals that *L*c is invariant across the full range of ionic strengths investigated (0 M to 1 M NaCl). In contrast, the average value of *L*p changes slightly between 0 mM and 25 mM NaCl, while that of *S* shows a modest, but clear rise as the concentration of NaCl is increased from 0 mM to 150 mM. These observations mirror similar findings for end-opened torsionally-relaxed DNA by Baumann *et al*. (2). Here, the authors suggested that the reduced values of *S* at lower NaCl concentrations could result from base-pair breathing at forces below 30 pN.

Using the parameters determined in Supplementary Fig. S5 (panels a–c), we calculated FD curves using the eWLC model (with 0.1 pN step-size). From these calculated curves, we then extracted the 2nd derivative using a Savitzky-Golay (20 point) smoothing filter (Supplementary Fig. 5d). Next, we determined the extension and force in the calculated FD curves at which T1 occurs as a function of NaCl concentration (Supplementary Fig. 5, panels e and f, respectively). A similar procedure was performed for the $MgCl_2$

concentration sweep, displayed in Supplementary Fig. 6. As appreciated from Supplementary Figs. 5 and 6 (panels e and f), changes in the extension and force of torsionally-constrained DNA at T1 can largely be explained by the perturbations to *L*p and *S* of the molecule as a function of ionic strength.

Peak intensities of T1–T4 as a function of ionic strength. The intensity of each peak identified in the 2nd derivative profiles of torsionally-constrained DNA (Fig. 2a and Supplementary Fig. 3a) is essentially determined by the difference in slope of FD curves before and after each transition (T1–T4). By plotting the peak intensity of T1–T4 as a function of ionic strength (Supplementary Fig. 7), we can determine how salt influences the change in pitch of FD curves during each transition.

Most notable here is the change in magnitude of the 2nd derivative peak at T4. Supplementary Fig. 7 reveals that the magnitude of this T4 peak is progressively larger as the ionic strength is increased. This change reflects two parameters: (i) the cooperativity of overstretching between T3 and T4 and (ii) the stiffness of the DNA molecule at forces > T4. As shown in Supplementary Fig. 8, the latter is largely constant as a function of either monovalent or divalent salt concentration. This indicates, therefore, that the increase in magnitude of the 2nd derivative peak at T4 as a function of ionic strength is primarily associated with an increasingly flat (and thus cooperative) overstretching plateau between T3 and T4. This in turn has the effect of further decreasing the force at which T4 occurs at high salt concentrations (as observed in Fig. 2e and Supplementary Fig. 3e).

Supplementary Methods

DNA construct design. λ-DNA (48,502 bp) was modified by ligating ‘end-cap’ oligomers to each end of the molecule. These oligomers were inspired by a design reported by Paik and Perkins (3), and consisted of a 5T loop, a 12 bp double-stranded stem, and a 12 nucleotide single-stranded overhang. The latter was complementary to either the left or right *cos* site of λ-DNA, respectively (4). Each end-cap was labelled with four biotin moieties. The general design of this DNA construct is shown in Supplementary Fig. 1.

Oligomer sequences. Below are the sequences for each end-cap ligated to λ-DNA. The boxes represent the four relevant domains of each oligomer, which from left to right are: the single-stranded overhang, one strand of the double-stranded stem, the 5T loop and the complementary strand of the double-stranded stem. The underlined nucleotides are labelled with a biotin.

End cap #1:

5’ AGG TCG CCG CCC | GGA GTT GAA CGT | TTTTT | ACG TTC AAC TCC 3’

End cap #2:

5’ GGG CGG CGA CCT | CAA GTT GGA CAA | TTTTT | TTG TCC AAC TTG 3’

DNA preparation protocol. λ-DNA (Roche) and the two end-caps (custom made by Biolegio) were first phosphorylated by reaction with polynucleotide kinase (NEB), as follows:

- Prepare phosphorylated λ-DNA (14 nM): λ-DNA (50 μl, 250 μg/ml) + 10x T4 DNA ligase buffer (5.5 μl, Fermentas) + polynucleotide kinase (0.5 μl, 10,000 units/ml)

- Prepare phosphorylated end-cap #1 (10 μM): end-cap #1 (2 μl, 100 μM) + de-ionized water (15.5 μl) + 10x T4 DNA ligase buffer (2 μl, Fermentas) + polynucleotide kinase (0.5 μl, 10,000 units/ml)

- Prepare phosphorylated end-cap #2 (10 μM): end-cap #2 (2 μl, 100 μM) + de-ionized water (15.5 μl) + 10x T4 DNA ligase buffer (2 μl, Fermentas) + polynucleotide kinase (0.5 μl, 10,000 units/ml)

These reactions were left for one hour at 37°C. Following phosphorylation, end-cap #1 was incubated with λ-DNA in ~10-fold excess, as follows:

- 10x T4 DNA ligase buffer (50 μl, Fermentas) + de-ionized water (400 μl) + phosphorylated λ-DNA (56 μl, 14 nM) + phosphorylated end-cap #1 (1μl, 10 μM)

To ensure that the end-cap annealed in its minimum energy configuration prior to the ligation, the above mixture was heated to 80°C for five minutes, before being cooled rapidly on ice. Next, T4 DNA ligase enzyme (4 μl, 200 units Fermentas) was added, and the solution was left to react at 16ºC for 12 hours.

Phosphorylated end-cap #2 (10 μl, 10 μM) was then added to the above solution (in ~100-fold excess to λ-DNA). The mixture was heated to 80°C for five minutes, and cooled rapidly on ice. T4 DNA ligase enzyme (4 μl, 200 units Fermentas) was subsequently added to the solution and left to react at 16ºC for 12 hours. Once completed, the ligated DNA construct was purified and extracted by ethanol precipitation and stored in TE buffer.

Preparation of end-closed torsionally-constrained DNA. Once each end-cap was ligated to λ-DNA, the DNA molecule could be tethered between two streptavidin-coated beads via biotin-streptavidin bonds. The number of biotins which bind to a given bead is variable: in the majority of cases (typically ~80%), more than two biotins on each end of the DNA molecule bind to a bead. This renders the DNA molecule unable to change its total linking number and the molecule is thus torsionally-constrained, as highlighted in Supplementary Fig. 2a.

Preparation of end-closed torsionally-relaxed DNA. Torsionally-relaxed DNA could be prepared using the DNA construct depicted in Supplementary Fig. 1, since in ~20% of cases only a single biotin moiety is successfully tethered to a bead on at least one end of the

DNA molecule. As a result, the DNA molecule is free to rotate around its axis (via the single biotin-streptavidin bond) as tension is applied (5). This assay is illustrated schematically in Supplementary Fig. 2b. Since the DNA molecule is still end-capped (*i.e*. contains no nicks or free-ends), the formation of peeled single-stranded DNA during overstretching is topologically forbidden (5). This allowed us to compare torsionally-constrained DNA with torsionally-relaxed DNA, while maintaining topological constraint (Figs. 1 and 4).

Supplementary References

[1] Rybenkov, V. V., Vologodskii, A. V. & Cozzarelli, N. R. The effect of ionic conditions on DNA helical repeat, effective diameter and free energy of supercoiling. *Nucleic Acids Res.* **25,** 1412-1418 (1997).

[2] Baumann, C. G., Smith, S. B., Bloomfield, V. A. & Bustamante, C. Ionic effects on the elasticity of single DNA molecules. *Proc. Nat. Acad. Sci. USA* **94,** 6185-6190 (1997).

[3] Paik, D. H. & Perkins, T. T. Overstretching DNA at 65 pN does not require peeling from free ends or nicks. *J. Am. Chem. Soc.* **133,** 3219-3221 (2011).

[4] Nichols, B. P. & Donelson, J. E. 178-nucleotide sequence surrounding the *cos* site of bacteriophage lambda DNA. *J. Virol.* **26,** 429-434 (1978).

[5] King, G. A. *et al*. Revealing the competition between peeled ssDNA, melting bubbles, and S-DNA during DNA overstretching using fluorescence microscopy. *Proc. Nat. Acad. Sci. USA* **110,** 3859-3864 (2013).
